# Anxiety and fear of COVID-19 as potential mechanisms to explain vaccine hesitancy among adults

**DOI:** 10.3389/fpsyt.2024.1376567

**Published:** 2024-05-03

**Authors:** Fahmi H. Fadhel, Nabil Saleh Sufyan, Mohammed M. J. Alqahtani, Ahmed Ali Almaamari

**Affiliations:** ^1^Psychology Program, Social Science Department, College of Arts & Sciences, Qatar University, Doha, Qatar; ^2^Psychology Department, College of Education, King Khalid University, Abha, Saudi Arabia; ^3^Psychology Department, College of Education, Qassim University, Qassim, Saudi Arabia

**Keywords:** anxiety, vaccine hesitancy, interrelationship, predicting factors, COVID-19

## Abstract

**Background:**

Vaccine hesitancy is a significant global problem resulting from the interaction of multiple factors, including mental health factors. However, the association of COVID-19 vaccine hesitancy with mental health has not been well-examined, especially in Arab culture. This study aims to identify the correlation between anxiety/fear of COVID-19 and vaccine hesitancy among Saudi adults.

**Methods:**

An online-based survey was administered to 558 participants from all regions of Saudi Arabia using the snowball technique. However, this sample may not be representative of the Saudi adult population. Participants responded to the Questionnaire of Vaccine Hesitancy, the COVID-19-Anxiety Questionnaire (C-19-A), and the Fear of COVID-19 Scale (FCV-19S). Data were analyzed on vaccine uptake, vaccine hesitancy, coronavirus infection, and demographic variables. The predictive factors of vaccine hesitancy were examined in one model using multiple regression analysis by the Enter method (*P=* 0.05).

**Results:**

COVID-19 anxiety and fear have significant correlations with vaccine hesitancy (*Phi*=0.33, *P*=0.017; *Phi*=0.29, *P*=0.013, respectively). Anxiety and fear were higher among unhesitating participants (t =2.469, *P*=0.014; t=2.025, *P*=0.043, respectively). Participants who had previously been infected with coronavirus were more likely to be hesitant (X^2 = ^23.126, P=0.000). Participants who scored high in anxiety were more likely to be vaccinated (F=3.979, P=0.019) and have a secondary school or college education (F=4.903 *P*=0.002). COVID-19 anxiety, gender, and coronavirus infection significantly predicted vaccine hesitancy.

**Conclusion:**

Anxiety and fear of COVID-19 are among the most important factors correlated with vaccine hesitancy; unhesitant people are more likely to have anxiety and fear. COVID-19 anxiety significantly predicted vaccine hesitancy. We recommend integrating psychological care into vaccination plans to help increase the uptake rate during potential subsequent pandemics. Relevant intervention programs can be designed to help increase vaccine acceptance, deal with vaccine hesitancy, and relieve psychological symptoms during major pandemics. Psychologists can provide awareness messages, counselling seminars, online mentoring, or telemental health outreach.

## Introduction

The Coronavirus continues to pose major challenges to public health globally, with the emergence of new variants that need further efforts to contain and manage the virus. In this case, vaccinations are an important way to limit the spread of the COVID-19 pandemic. However, vaccine hesitancy is one of the most important and influential issues impeding efforts to contain the coronavirus; it is associated with other factors that have a direct or indirect impact on vaccine uptake, including psychological factors (1, 2). However, the association of COVID-19 vaccine hesitancy with mental health has not been well-examined in the Middle East and Arab countries. Therefore, it is important to identify these psychological factors to derive interventions and promote vaccine acceptance (3).

Several studies have found, during the COVID-19 pandemic, an increased prevalence of mental health disturbances, particularly anxiety, fears, and depression, among the general public or healthcare workers (1, 4). Although useful, the results of these studies did not go beyond the outbreak of the epidemic to the stage of vaccination, they addressed mental health disturbances without making a direct link to COVID-19 vaccine hesitancy, and their ability to explain why some individuals are reluctant to vaccinate is still limited (5).

Consistently, researchers have indicated that the observed increase in symptoms of anxiety and depression reported during the COVID-19 pandemic could have implications for vaccine hesitancy (6). However, there is a significant discrepancy in the results of previous studies that addressed this issue. For example, participants who reported feeling anxious, sad, and agitated every day due to social restrictions had high vaccine hesitancy, while those who reported the same feelings on only some days were less hesitant (7). Other studies documented that those reporting symptoms of anxiety or depression were less vaccine-hesitant (5).

Although it appears that anxiety, fear, and other psychological disorders are among the causes of vaccine hesitancy, the relationship between mental disorders and hesitation may be reciprocal. Concerns about the safety and effectiveness of the vaccine, and possible side effects, trigger vaccine hesitancy and resistance. Therefore, hesitant individuals are in direct confrontation with society and, as a result, are exposed to more stress and anxiety, the anticipation of potential risks, and other forms of psychological distress. Consistent with this point, it was found that individuals with a high risk of developing depression were more concerned about the efficacy and safety of the COVID-19 vaccine, which made them more reluctant to get vaccinated (8). Therefore, the most informative and effective approach in this area may be to recognize the underlying mechanisms of psychological morbidity that distinguish individuals who are reluctant to receive the vaccine from those who are not (5) – an approach based on studying the association of psychological symptoms with vaccine hesitancy. However, studies of mental health and its association with vaccine hesitancy are scarce; the evidence of significant correlations between symptoms of psychological disturbances and hesitation also remains limited (8), especially in the Middle East and Arab countries.

Given these gaps in the literature, this study aims to identify (1) the relationship between anxiety/fear of COVID-19 and vaccine hesitancy, (2) the relationship between COVID-19 infection and vaccine hesitancy, and (3) the degree to which COVID-19 anxiety and fear of COVID-19 predict vaccine hesitancy.

## Methods

### Participants and recruitment procedures

This study was based on the survey method to assess the relationship between COVID-19 anxiety/fear of COVID-19 and vaccine hesitancy. The survey was conducted online in the Arabic language in July 2021 and relied on self-reported data from a Saudi population who had already been offered vaccination (9). Participants were recruited online using the snowball technique, as the questionnaire was advertised in psychotherapy and mental health forums in Saudi Arabia. The questionnaire was sent via SMS message or email to individuals who provided their phone numbers or email addresses. Of the 2,113 individuals who were contacted via phone message or email, 558 participated in this study (with a response rate of 27.44%). All 558 respondents fully completed the questionnaire and there are no missing data in this study. However, the sample of this study may not be representative of the Saudi adult population.

### Ethical considerations

All procedures in this study were consistent with the ethical standards of the 1964 Declaration of Helsinki or similar ethical standards. Participation in this research was voluntary, and the participants provided informed consent. The inclusion criteria included express written consent; a minimum age of 18 years; Arabic language proficiency; and current residency in Saudi Arabia.

### Measures

#### Demographics

Demographic variables include (a) age, gender, education level, and marital status; and (b) vaccine-related variables including vaccine uptake, number of vaccine doses, vaccine hesitancy, and whether the participant was previously infected with the coronavirus. Age was classified into three gropes; young adults (18-37), middle-aged adults (38-47), and older adults (48-65) years. Gender was coded as “male” vs. “female.” Education level was coded as “secondary school,” “college graduate,” and “higher degree holders.” Marital status was classified as “single,” married, and “divorced/widowed.” Vaccine uptake was classified into three groups; “vaccinated,” “unvaccinated,” and “not decided yet”.

#### Vaccine hesitancy questionnaire

This questionnaire consists of four items designed in Arabic to assess COVID-19 vaccine hesitancy and related variables (e.g., COVID-19 infection, whether or not the person was vaccinated with a COVID-19 vaccine or was still hesitating, and the number of doses received if they were vaccinated). The response options are (“yes,” “no,” and “not sure”). Vaccine hesitancy was assessed with responses of “no” or “not sure”, while vaccine willingness was assessed with responses of “yes”.

#### COVID-19-anxiety questionnaire

The 10-item questionnaire was developed by Petzold et al. (10) to assess COVID-19 anxiety. The response options include five points on the Likert scale (1= ”strongly disagree,” 2=“disagree,” 3=“not sure,” 4=“agree,” and 5 = ”strongly agree”). The total score ranges from 10 to 50. The higher the overall score, the greater the anxiety. Petzold et al. (10) examined the psychometric properties of the scale and found good validity and reliability coefficients.

#### Fear of COVID-19 scale

This 7-item scale was developed by Ahorsu and his colleagues (11) to assess fear related to the coronavirus. The response options are on a 5-point Likert scale (1=“strongly disagree” to 5=“strongly agree”). The total score ranges from 7 to 35. The higher the overall score, the greater the fear of the coronavirus. This scale is valid, and its psychometric properties have been confirmed in several cultures (11).

In this study, we translated and validated the C-19-A and the FCV-19S into Arabic. The back-translated items were verified by two independent language experts who were bilingual in Arabic and English. The reliability of the scales was also examined in this study using Cronbach’s alpha. The scales showed good internal consistency (α=0.91) in the C-19-A, (α=0.91) in the FCV-19S, and (α=0.60) in the questionnaire of vaccine hesitancy. The Arabic versions of these scales are shown in the **Appendix A**.

### Data analysis plan

Statistical analysis was conducted with IBM SPSS software (version 25). Descriptive analysis was calculated for all demographic variables (i.e., age, gender, education, and marital status) and vaccine-related variables (e.g., vaccination, number of vaccine doses, vaccine hesitation, and whether the participant was previously infected with the coronavirus). Pearson’s chi-square test (X^2^) was used to investigate the relationship between COVID-19 infection and vaccine uptake. The relationship between COVID-19 anxiety/fear of COVID-19 and vaccine hesitancy was calculated by the “Phi” coefficient. ANOVA and t-tests were used to examine the differences between participants in terms of anxiety and fear of COVID-19 according to demographic and vaccine-related variables (i.e., gender, age groups, education level, marital status; vaccine uptake, number of vaccine doses, and vaccine hesitancy). In the regression analysis, vaccine hesitancy was treated as a dependent variable and the predictive factors (COVID-19 anxiety, fear of COVID-19, demographics, and previous infection with the coronavirus) of vaccine hesitancy were examined in one model using multiple regression analysis by the Enter method (*P=* 0.05). The variables of age, gender, education, marital status, and coronavirus infection were included in the statistical analysis as categorical variables. These independent variables were entered into the analysis one by one, and the final model was obtained based on retaining the significant variables in the analysis (P= 0.05).

## Results

**Table 1** presents the descriptive statistics of the sample (n= 558). The mean age was 38.66 ± 9.067 years (ranging from 18 to 65) and 53.8% of respondents were men. The majority of respondents (79%) indicated vaccine willingness, while 21% showed vaccine hesitancy.

**Table 1:** Descriptive Statistics of Demographic and Vaccine-Related Variables.

Gender	Male		Female								
	N	%	N	%							
	300	53.76	258	46.24							
	Mean	Std. D	Mean	Std. D	T		P-Value	Eta			
COVID-19 anxiety	29.34	8.51	28.32	9.28	1.345		0.179	0.058			
Fear of COVID-19	15.85	6.34	15.14	5.81	1.366		0.173	0.057			
Age groups	18-37 years		38-47 years		48-65 years						
	N	%	N	%	N	%					
	223	40	255	45.7	80	14.3					
	Mean	Std. D	Mean	Std. D	Mean	Std. D	F	*P-Value*	Eta		
COVID-19 anxiety	28.36	8.68	29.38	9.26	28.69	8.18	0.787	0.456	0.053		
Fear of COVID-19	14.93	5.95	15.96	6.43	15.74	5.34	1.765	0.172	0.080		
Marital status	Single		Married		Divorced/widowed						
	N	%	N	%	N	%					
	119	21.3	412	73.8	27	4.8					
	Mean	Std. D	Mean	Std. D	Mean	Std. D	F	*P-Value*			
COVID-19 anxiety	26.62	8.99	29.52	8.80	28.95	8.19	5.009	0.007	Eta		
Fear of COVID-19	13.92	5.74	15.93	6.08	16.37	6.85	5.373	0.005	0.138		
Education level	Secondary school		Diploma		Graduates		Higher education		0.133		
	N	%	N	%	N	%	N	%			
	51	9.14	123	22	325	58.2	59	10.6			
	Mean	Std. D	Mean	Std. D	Mean	Std. D	Mean	Std. D	F	*P-Value*	Eta
COVID-19 anxiety	30.57	9.25	26.72	9.25	29.74	8.73	27.14	7.62	4.903	0.002	0.108
Fear of COVID-19	16.24	6.72	14.52	6.40	15.93	5.99	14.73	5.31	2.165	0.091	0.161
COVID-19 Infection		Vaccinated		Unvaccinated		X^2^	*P-Value*		Eta		
		N	%	N	%						
	Yes	107	24.15	54	46.96	23.126	0.000		0.204		
	No	336	75.85	61	53.04						
	Total	443	79.3	115	21						

As per **Table 1**, out of 443 (79.39%) vaccinated participants, 107 (24.15%) had previously been infected with the coronavirus, compared to 54 (46.95%) among the non-vaccinated. The differences in COVID-19 anxiety according to marital status and education levels were significant. COVID-19 anxiety was higher among married participants (F=5.009, *P=* 0.007), and among those who have a secondary school or college education (F=4.903, *P=* 0.002). Participants who had high fear of COVID-19 were more likely to be divorced (F=5.373, *P=* 0.005), while the differences in fear of COVID-19 according to education level were not significant (F=2.156, *P=* 0.091). The differences in anxiety and fear of COVID-19 were not significant according to the variables of age (F=0.787, *P=* 0.456; F= 1.765, *P=* 0.172, respectively), gender (t=1.345, *P=* 0.179; t=1.366, P< 0.173, respectively), and previous infection with the coronavirus (t=0.040, *P=* 0.968; t=1.913, *P=* 0.056, respectively).

**Table 2** presents the differences in Anxiety/fear of COVID-19 according to Vaccine-Related Variables.

**Table 2:** The differences in Anxiety/fear of COVID-19 according to Vaccine-Related Variables.

Vaccine uptake	Vaccinated		Unvaccinated		Not decided yet		F	P-Value
	N	%	N	%	N	%		
	443	79.39	92	16.49	23	4.12		
	Mean	Std. D	Mean	Std. D	Mean	Std. D		
**COVID-19 anxiety**	29.35	8.63	26.50	9.72	29.30	9.15	3.97	0.019
**Fear of COVID-19**	15.79	6.06	14.40	6.33	14.87	5.66	2.10	0.123
**Number of vaccine doses**	**One shot**		**Two shots**					
	N	%	N	%				
	327	58.6	116	20.78				
	**Mean**	**Std. D**	**Mean**	**Std. D**	**T**	***P-Value***		
**COVID-19 anxiety**	29.20	8.859	29.74	7.95	-0.575	0.566		
**Fear of COVID -19**	15.46	5.97	16.68	6.23	1.857	0.064		
**COVID-19 infection**	**Yes**		**No `**					
	**Mean**	**Std. D**	**Mean**	**Std. D**	**T**	***P-Value***		
**COVID-19 anxiety**	28.85	9.57	28.88	8.59	0.04	0.968		
**Fear of COVID-19**	14.74	6.24	15.83	6.03	1.91	0.056		

As can be seen in **Table 2**, the differences in COVID-19 anxiety according to vaccine uptake (vaccinated, unvaccinated, and those who have not decided) were significant (F=3.979, *P=* 0.019). Vaccinated participants were more anxious than unvaccinated participants, while the differences in fear of COVID-19 according to vaccine uptake were not significant (F=2.102, *P=* 0.123). Meanwhile, the differences in anxiety and fear of COVID-19 according to COVID-19 infection were not significant (t= 0.040, P=0.968; t=1.913, P=0.056, respectively).

### Correlations between anxiety/fear of COVID-19 and vaccine-related variables

Phi correlation (Phi) showed significant correlations between anxiety**/**fear of COVID-19 and vaccine hesitancy, as shown in **Table 3**:

**Table 3:** Correlation of COVID-19 Anxiety and Fear of COVID-19 with Vaccine related variables.

Vaccine Hesitancy	Hesitant		Unhesitant		T	P-Value	Phi*	P-Value
	N	%	N	%				
	115	20.6	443	79.4				
	Mean	Std. D	Mean	Std. D				
COVID-19 anxiety	27.06	9.63	29.35	8.63	2.47	0.014	0.33	0.017
Fear of COVID-19	14.49	6.18	15.79	6.06	2.025	0.043	0.29	0.013

* Phi, phi correlation coefficient.

**Figure 1:**
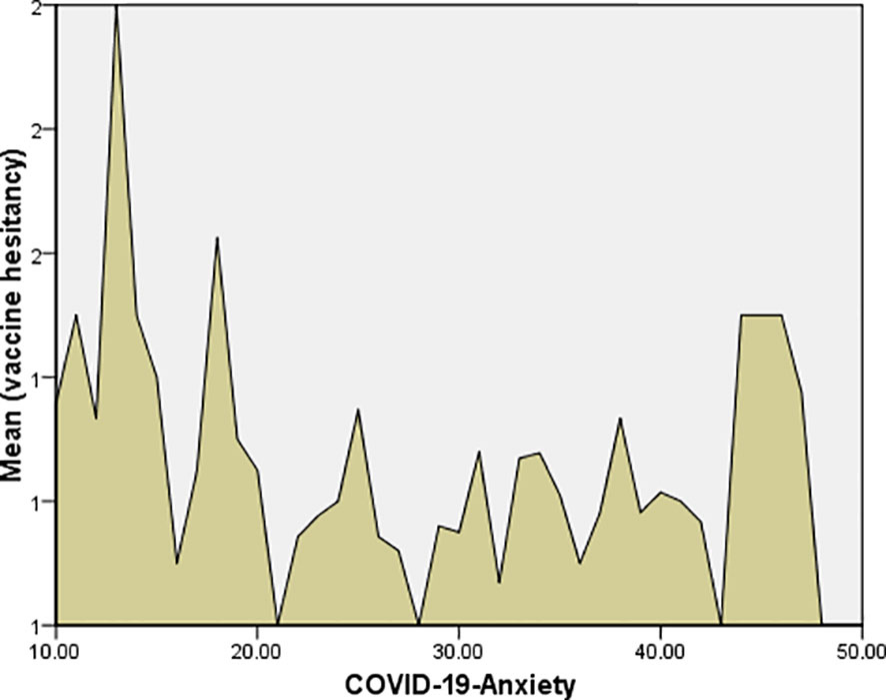
Correlation between COVID-19 anxiety and vaccine hesitancy.

**Figure 2:**
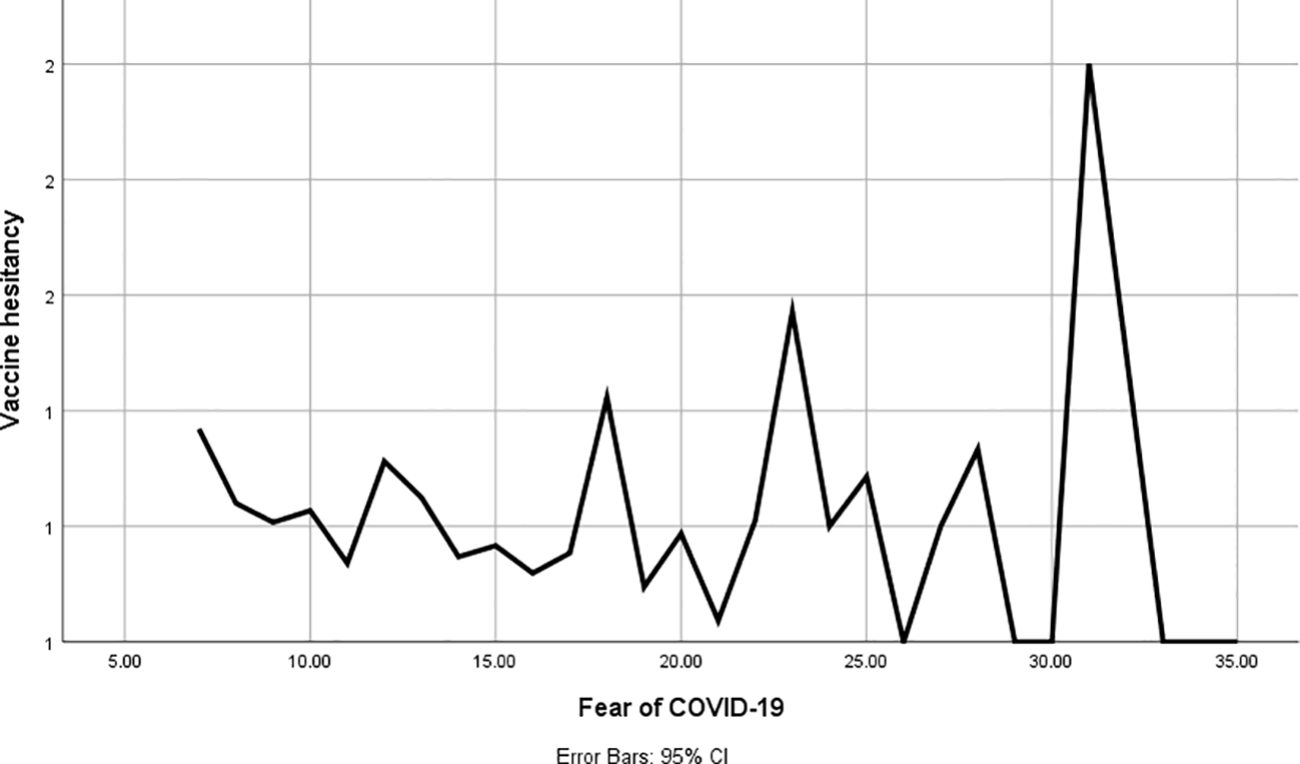
Correlation between the fear of COVID-19 and vaccine hesitancy.

As per **Table 3**, the correlations between COVID-19 anxiety and fear of COVID-19 with vaccine hesitancy were significant (*Phi*=0.33, *P*=0.017; *Phi*=0.29, *P*=0.013). In addition, COVID-19 anxiety and fear of COVID-19 were higher among unhesitating participants, and the differences were significant (t= 2.469, P=0.014; t= 2.025, P=0.043, respectively), as shown in **Figures 1** and **2**.

Meanwhile, participants who had previously been infected with COVID-19 were more likely to be hesitant, and the differences were significant (X^2 = ^23.126, *P=* 0.000).

### Degree to which COVID-19 anxiety and fear of COVID-19 predict vaccine hesitancy

The predicting factors of vaccine hesitancy include COVID-19 anxiety, gender, and COVID-19 infection. These results are presented in **Table 4**.

**Table 4:** Linear regression analysis for predictors of vaccine hesitancy.

Model Summary						
Model	R	R Square	Adjusted R Square		Std. Error of the Estimate	
**1**	.278^a^	.077	.065		0.391	
	**ANOVA^a^**					
		**Sum of Squares**	**df**	**Mean Square**	**F**	***P-Value***
	Regression	7.043	7	1.006	6.568	0.000^b^
	Residual	84.256	550	.153		
	Total	91.299	557			
		**B**	Std. Error	**Beta**	**T**	***p-Value***
	(Constant)	1.748	.133		13.167	.000
	Age groups	-.038-	.027	-.065-	-1.42-	.157
	Gender	.070	.034	.087	2.064	.040
	Education	-.042-	.021	-.081-	-1.954	.051
	Marital status	-.022-	.037	-.026-	-.574-	.567
	Covid-19 infection	-.177-	.037	-.198-	-4.780	.000
	Covid-19 anxiety	-.006-	.003	-.141-	-2.129	.034
	Fear of Covid-19	.003	.004	.045	.683	.495

a. Dependent Variable: vaccine hesitancy. b. Predictors: (Constant), age, gender, education, marital status, COVID-19 infection, COVID-19-Anxiety, and Fear of COVID-19.

As per **Table 4**, COVID-19 anxiety, gender, and previous coronavirus infection were significant predicted COVID-19 vaccine hesitancy.

## Discussion

The present study examined the correlation between COVID-19 anxiety/fear of COVID-19 and vaccine hesitancy. The findings indicated that anxiety and fear of COVID-19 significantly explained vaccine hesitancy among Saudi adults. Anxiety and fear were high among unhesitating participants. Likewise, vaccinated people were more anxious than unvaccinated people, while there were no significant differences between vaccinated and unvaccinated participants regarding fear of COVID-19. These results should be reasonable, increased COVID-19 anxiety among vaccinated participants may be partly attributed to concerns about the efficacy and safety of the vaccine (2). It is expected that the side effects of COVID-19 vaccines will be higher among vaccinated people. As a result, unvaccinated persons may have fewer concerns regarding vaccine safety and effectiveness, which partly contributed to the significant differences between vaccinated and unvaccinated participants in the present findings.

Moreover, COVID-19 anxiety, gender, and previous infection with the Coronavirus statistically significantly predicted vaccine hesitancy, while COVID-19 fear and the variables of education and marital status did not predict vaccine hesitancy.

Although the regression model in **Table 4** indicates relatively low values in the R-squared value at 0.077 and the adjusted R-squared value at 0.065, the total F value at (6.568) was high and significant at the 0.001 level, and this significance was reversed on the prediction values of the COVID-19 anxiety, gender, and previous infection with the Coronavirus that significantly predicted vaccine hesitancy. These results demonstrate the importance of the model despite the observed weaknesses which may be attributed to the role of variation among participants in the variables of fear of COVID-19, age, education, and marital status, as evidenced by the low predictive value of these demographic variables in explaining vaccine hesitancy in the current model. We acknowledge a limitation regarding the modest R-squared and adjusted R-squared values reported in our study, we have undertaken a comprehensive evaluation of our model’s capacity to account for variance in vaccine hesitancy. These values, while on the lower end of the spectrum, reflect the complex and multifactorial nature of vaccine hesitancy as a behavioral phenomenon. Despite the apparent limitations suggested by the R-squared values, the statistical significance of the predictors identified underscores their importance in understanding vaccine hesitancy.” Further studies will be urgently needed to clarify these effects.

On the other hand, these results are consistent with the results of previous studies that found that mental health problems are among the predictive factors of vaccine hesitancy (12, 13). Wang et al. (14) found that generalized anxiety disorder increased directly with vaccine hesitancy. Studies also have documented higher levels of psychological problems related to COVID-19 vaccinations, including anxiety, fear, and suicidal thoughts (15, 16). People with high levels of worry or anxiety were less hesitant about the COVID-19 vaccine, and vice versa (17). Other studies reported a significant correlation between fear of COVID-19 and vaccine hesitancy; the higher the fear of COVID-19, the lower the vaccine hesitancy (18, 19). Researchers also realize that people with anxiety or depression symptoms are less vaccine-hesitant (5). A total of 40% of those vaccinated against influenza indicated that they feared contracting COVID-19 (20), and the relationship between anxiety/fear of COVID-19 and vaccine acceptance was significant (3).

The significant correlations between COVID-19 anxiety/fear of COVID-19 and vaccine hesitancy that revealed in our results and indicated that anxiety and fear were high among unhesitating participants does not necessarily reflect a causal relationship between vaccine hesitancy and these psychological factors. Other factors may have played an important role in this relationship, including cultural variables and the social context in the Middle East and Arab countries, which led to the contradiction between our results and the results of some previous studies.

Contrary to the results of the current study, research found that the absence or presence of little fear of COVID-19 was significantly associated with vaccine hesitancy (21), and lower levels of depression were significantly associated with vaccine acceptance (22), while lower anxiety was associated with vaccine hesitancy (3, 19). Meanwhile, psychological distress was higher among COVID-19 vaccine-hesitating individuals (23), while a pre-pandemic diagnosis of anxiety, depression, or distress was unrelated to vaccine uptake (24). In addition, people who reported anxiety and anger during the lockdown and social restrictions were more hesitant, while those who experienced these feelings at specific periods were less hesitant (7).

The relationship between fear of COVID-19 and vaccine acceptance were not significant (3). Other studies did not find a significant relationship between COVID-19 vaccine hesitancy and mental health (25).

Our findings showed that COVID-19-Anxiety, gender, and coronavirus infection were significant predictors of vaccine hesitancy. Moreover, the correlation between COVID-19 anxiety and vaccine hesitancy was significant, indicating that an increase in hesitation corresponds with a decrease in COVID-19 anxiety, and vice versa. These results suggest that COVID-19 anxiety and fear may be among the reasons for receipt of the vaccine. Therefore, the higher the anxiety (or fear), the lower the vaccine hesitancy. We believe that anxiety is forcing people to protect themselves through vaccines. However, most causes of this phenomenon remain complex and unclear, as they include social, demographic, psychological, behavioral, and cultural factors (1, 2). Therefore, understanding the psychological factors that explain vaccine hesitancy is important for psychologists, healthcare workers, and authorities to improve vaccine acceptance. As reported in prior studies (1–4), psychological variables play a prominent role during the COVID-19 pandemic.

Consequently, it would be helpful to use these psychological variables to enhance the uptake rate in the future. In line with our results, lower level of fear and worsening health status were the predictive factors of vaccine hesitancy (7, 21). Prior studies indicated that anxiety, fears, and perceived psychological state had positive indirect effects on vaccine hesitancy (3, 26). Another study found a significant indirect effect of psychological distress on vaccine hesitancy via other factors, including risk perception and vaccine distrust (8). Predictive factors of vaccine willingness include fear of COVID-19 (27), age (2), more concerns about COVID-19, and few concerns about the vaccination (28). Additionally, anxiety, the fear of COVID-19, and perceived COVID-19 infectability were associated significantly with (3, 27).

The findings of the current study showed that coronavirus infection had a significant effect on vaccine hesitancy, as those who were previously infected were more hesitant than those who had never been infected with the coronavirus. These results may be partly attributed to the fact that those who have been infected with the coronavirus (particularly during the first wave of the pandemic) believe that they had acquired immunity against COVID-19, which will protect them from further infections in the future. In line with these findings, Hwang et al. (21) found that participants who perceived little risk of COVID-19 were more likely to be hesitant.

On the Contrary, studies showed that the lower perceived risk of coronavirus infection was associated with vaccine hesitancy (3, 7, 29). Participants who had previously been tested for COVID-19 were less likely to be vaccine-hesitant (30). The discrepancy between the results of these studies and our results may be due to methodological differences or to the differences in cultural and social contexts in which these studies were conducted.

The results of the present study showed that anxiety and fear of COVID-19 differ according to educational level in that they were higher among persons with a secondary school or college education than among persons with higher education, while COVID-19 anxiety was higher among married people. Meanwhile, participants who had high fear of COVID-19 were more likely to be divorced. These results may be because married people worry about their families contracting the coronavirus and because those with low educational levels are more likely to accept misinformation about the coronavirus and vaccination, as it was found that fear of COVID-19 decreased with higher levels of education (20). Increased fear of COVID-19 among divorced participants may be attributed to concerns about infection, particularly when alone (31). Contrary to the findings of the current study, the risk of anxiety was lower among married people (32).

Our findings provide important evidence about a potential mechanism regarding the significant effects of COVID-19 anxiety and fear of COVID-19 on vaccine hesitancy. These results present the importance of studying the association between psychological factors and vaccine hesitancy during major pandemics. Follow-up studies may be needed to evaluate symptoms of COVID-19 anxiety and fear after vaccinations, especially because it was pointed out that the first dose of the COVID-19 vaccine led to a significant improvement in the mental health of adults (33).

## Conclusions

The results of this study indicate that anxiety and fear of COVID-19 are among the most important factors related to vaccine hesitancy; people who have received the coronavirus vaccine are more likely to have anxiety and fear, while anxiety and fear were high among unhesitating participants. Our results showed that COVID-19 anxiety and fear of COVID-19 differ according to the variables of marital status and educational level in that COVID-19 anxiety was high among married people and among those with secondary or university degrees. While the fear of COVID-19 was high among divorced individuals.

COVID-19-Anxiety, gender, and previous coronavirus infection were significant predictors of vaccine hesitancy. Participants who were previously infected were more hesitant than those who had never been infected with the coronavirus. Our findings could be valuable in a wide-ranging and better understanding of the psychological impacts of the COVID-19 pandemic. Therefore, we recommend integrating psychological care into vaccination plans in the future. Relevant intervention programs can be designed to help increase vaccine acceptance, deal with vaccine hesitancy, and relieve psychological symptoms during major pandemics. Psychologists can provide awareness messages, seminars and psychological counselling posters, or teletherapy outreach. Research evaluating the associations between psychological symptoms and vaccine hesitancy is highly needed to improve psychotherapy and mental health care planning during potential subsequent pandemics.

### Strengths and limitations

The strength of the current study is that it seeks to reveal the relationship between anxiety/fear of COVID-19 and vaccine hesitancy, especially given that few studies exist in this area. However, this study has some limitations. In general, reliance on the self-report questionnaire has many shortcomings and deficiencies. Also, the non-representative sample of the Saudi community is a limitation that can be overcome in the methodologies of similar studies in the future. These limitations hinder the possibility of generalizing the results of the current study to the general adult population in the KSA. Moreover, the interactions of vaccination with anxiety and fear of COVID-19 may arise from one or several mechanisms. These effects are likely to be diverse and depend on characteristics beyond those analyzed in this study. In addition, the fact that people who receive the vaccines at different times differ in several psychological and socio-demographic characteristics suggests that the effects may differ from those of individuals vaccinated after the period of this study, as noted by Perez-Arce et al. (33). It is advisable to go beyond the limitations of the current research in similar prospective studies.

## Data availability statement

The raw data supporting the conclusions of this article will be made available by the authors, without undue reservation.

## Ethics statement

The studies involving humans were approved by The Research Ethics Committee at King Khalid University. The studies were conducted in accordance with the local legislation and institutional requirements. The participants provided their written informed consent to participate in this study.

## Author contributions

FF: Conceptualization, Data curation, Formal analysis, Funding acquisition, Investigation, Methodology, Project administration, Resources, Software, Supervision, Validation, Visualization, Writing – original draft, Writing – review & editing. NS: Conceptualization, Data curation, Investigation, Methodology, Project administration, Resources, Writing – original draft. MA: Data curation, Methodology, Project administration, Resources, Writing – original draft. AA: Data curation, Methodology, Project administration, Resources, Conceptualization, Investigation, Writing – review & editing.
