# Supplementary material for: Anxiety and fear of COVID-19 as potential mechanisms to explain vaccine hesitancy among adults
Source: Front Psychiatry. 2024 May 3;15:1376567. doi: 10.3389/fpsyt.2024.1376567 (PMC11099610; doi:10.3389/fpsyt.2024.1376567)
Supplement: Supplementary file 1 [file DataSheet_1.docx]

Appendix A: Arabic version of the scales:

1. **Table 1. The Questionnaire of Vaccine Hesitancy**

| **رقم** | **العبارات** | **الاجابات** | | |
| --- | --- | --- | --- | --- |
| 1 | هل أخذت لقاح كورونا؟ | نعم أخذت لقاح كورونا | لا لم اخذ اللقاح حتى الآن | ما زلت متردد عن اخذ اللقاح |
| 2 | سواء كنت قد أخذت أو لم تأخذ لقاح كورونا حتى الآن، هل أنت مستعد وموافق على أخذ اللقاح؟ | نعم مستعد، وموافق | لا لست مستعد ولست موافق على أخذ اللقاح الآن | لست متأكد |
| 3 | كم جرعة أخذت من لقاح كورونا حتى الآن؟ | جرعة واحدة | جرعتان | لم اخذ اللقاح حتى الآن |
| 4 | هل سبق لك أن رفضت أو ترددت في الحصول على أي لقاح من قبل (مثل لقاح الإنفلونزا)؟ | نعم | لا | لست متأكد |

1. **Table 2. The Arabic version of the** **COVID-19-Anxiety** **Questionnaire (C-19-A)**

| **رقم** | **خلال جائحة فيروس كورونا:** | **موافق بشدة**  **5** | **موافق 4** | **أحيانا 3** | **غير موافق 2** | **غير موافق بشدة 1** |
| --- | --- | --- | --- | --- | --- | --- |
| 1 | شعرت بالذعر أو الخوف أو برعب مفاجئ بسبب كورونا |  |  |  |  |  |
| 2 | شعرت بالقلق أو الخوف أو التوتر بسبب كورونا |  |  |  |  |  |
| 3 | راودتك أفكار عن التعرض للإصابة بكورونا، أو صعوبة التغلب على الخوف، أو أن أشياء سيئة أخرى ستحدث بسبب كورونا |  |  |  |  |  |
| 4 | شعرت بتسارع ضربات القلب، أو التعرق، أو صعوبة التنفس، أو الإغماء، أو الارتعاش بسبب كورونا |  |  |  |  |  |
| 5 | شعرت بتوتر في العضلات، أو شعرت بالضيق أو القلق، أو واجهتك صعوبة في الاسترخاء بسبب كورونا |  |  |  |  |  |
| 6 | تجنبت، أو لم تدخل بعض الأماكن، أو المواقف، أو لم تقترب منها بسبب كورونا |  |  |  |  |  |
| 7 | ابتعدت عن موقف، أو عن مكان ما، أو خرجت منه مبكرا |  |  |  |  |  |
| 8 | تستغرق كثير من الوقت في الاستعداد للذهاب لمناسبة ما، أو تماطل في الذهاب إليها |  |  |  |  |  |
| 9 | تشغل نفسك أو تلهيها باي شيء لكي تتجنب التفكير في كورونا |  |  |  |  |  |
| 10 | احتجت إلى مساعدة للتعامل مع بعض المواقف أو الأحداث أو الأشخاص |  |  |  |  |  |

1. **Table 3. The Arabic version of the Fear of COVID-19 Scale (FCV-19S)**

| **رقم** | **الفقرة** | **غير موافق بشدة**  **1** | **غير موافق**  **2** | **بين بين**  **3** | **موافق**  **4** | **موافق بشدة**  **5** |
| --- | --- | --- | --- | --- | --- | --- |
| 1 | أنا أكثر خوفًا من فيروس كورونا |  |  |  |  |  |
| 2 | أشعر بعدم الارتياح عندما أفكر في فيروس كورونا |  |  |  |  |  |
| 3 | تصبح يدي رطبة أو مبتلة بالعرق عندما أفكر في فيروس كورونا |  |  |  |  |  |
| 4 | أخشى أن أفقد حياتي بسبب فيروس كورونا |  |  |  |  |  |
| 5 | أشعر بالتوتر أو القلق عندما اتابع الأخبار أو القصص عن فيروس كورونا |  |  |  |  |  |
| 6 | لا أستطيع النوم لأنني قلق بشأن الإصابة بفيروس كورونا |  |  |  |  |  |
| 7 | قلبي يتسارع أو يخفق بشدة عندما أفكر في إصابتي بفيروس كورونا |  |  |  |  |  |

**Table 4. The Questionnaire of Vaccine Hesitancy (4-items).*.**

| Items |  |  |  |  |
| --- | --- | --- | --- | --- |
| 1 | Have you received a COVID-19 vaccine? | **Yes** | **No** | I'm still hesitant about taking the vaccine |
| 2 | Whether or not you had gotten the COVID-19 vaccine, are you ready and agree to receive the vaccine | **Yes** | **No** | Not sure |
| 3 | How many doses of the COVID-19 vaccine you had taken? | **One dose** | **Two dose** | I haven't taken the vaccine yet |
| 4 | Had you refused a vaccination—such as the influenza vaccine—in the past? | **Yes** | **No** | I'm not sure |

* We evaluated COVID-19 vaccine hesitancy with the question, “Have you received a COVID-19 vaccine?”

**Table 5. The COVID-19-Anxiety Questionnaire (10-items).**

| **N** | **Item**  . | **Strongly agree** | | **Agree** | | **Not sure** | | **Disagree** | | **Strongly disagree** | |
| --- | --- | --- | --- | --- | --- | --- | --- | --- | --- | --- | --- |
|  |  |  |  |  |  |  |  |  |  |  |  |
| 1 | Felt moments of sudden terror, fear, or fright due to Corona |  |  |  |  |  |  |  |  |  |  |
| 2 | Felt regarding Corona anxious, worried, or nervous. |  |  |  |  |  |  |  |  |  |  |
| 3 | Had thoughts of being injured, overcome with fear, or other bad things happening due to Corona. |  |  |  |  |  |  |  |  |  |  |
| 4 | Due to anxiety regarding Corona, I felt a racing heart, sweaty, trouble breathing, faint, or shaky in these situations. |  |  |  |  |  |  |  |  |  |  |
| 5 | Felt tense muscles, felt on edge or restless, or had trouble relaxing due to Corona. |  |  |  |  |  |  |  |  |  |  |
| 6 | Avoided, or did not approach or enter, situations linked with Corona. |  |  |  |  |  |  |  |  |  |  |
| 7 | Moved away from these situations or left them early. |  |  |  |  |  |  |  |  |  |  |
| 8 | Spent a lot of time preparing for, or procrastinating about (i.e., putting off), situations linked to Corona. |  |  |  |  |  |  |  |  |  |  |
| 9 | Distracted myself to avoid thinking about Corona. |  |  |  |  |  |  |  |  |  |  |
| 1 | Needed help to cope with these situations (e.g., alcohol or medications, superstitious objects, other people). |  |  |  |  |  |  |  |  |  |  |

**Table 6. The Fear of COVID-19 Scale (7-items).**

| **N** | **Item**  . | **Strongly agree** | | **Agree** | | **Not sure** | | **Disagree** | | **Strongly disagree** | |
| --- | --- | --- | --- | --- | --- | --- | --- | --- | --- | --- | --- |
|  |  |  |  |  |  |  |  |  |  |  |  |
| 1 | I am most afraid of coronavirus. |  |  |  |  |  |  |  |  |  |  |
| 2 | It makes me uncomfortable to think about coronavirus. |  |  |  |  |  |  |  |  |  |  |
| 3 | My hands become clammy when I think about coronavirus. |  |  |  |  |  |  |  |  |  |  |
| 4 | I am afraid of losing my life because of coronavirus |  |  |  |  |  |  |  |  |  |  |
| 5 | When watching news and stories about coronavirus-19 on social media, I become nervous or anxious. |  |  |  |  |  |  |  |  |  |  |
| 6 | I cannot sleep because I am worrying about getting coronavirus. |  |  |  |  |  |  |  |  |  |  |
| 7 | My heart races or palpitates when I think about getting coronavirus. |  |  |  |  |  |  |  |  |  |  |

**Appendix B. Response rate to the items of the COVID-19-Anxiety Questionnaire (10-items).**

| **N** | **Item**  . | **Strongly agree** | | **Agree** | | **Not sure** | | **Disagree** | | **Strongly disagree** | |
| --- | --- | --- | --- | --- | --- | --- | --- | --- | --- | --- | --- |
|  |  | **N** | **%** | **N** | **%** | **N** | **%** | **N** | **%** | **N** | **%** |
| 1 | Felt moments of sudden terror, fear, or fright due to Corona | 56 | 10 | 99 | 17 | 185 | 33.2 | 118 | 21 | 100 | 17.9 |
| 2 | Felt regarding Corona anxious, worried, or nervous. | 51 | 9 | 78 | 14 | 183 | 32.8 | 139 | 25 | 107 | 19.2 |
| 3 | Had thoughts of being injured, overcome with fear, or other bad things happening due to Corona. | 60 | 10.8 | 114 | 20.4 | 185 | 33.2 | 119 | 21.3 | 80 | 14.3 |
| 4 | Due to anxiety regarding Corona, I felt a racing heart, sweaty, trouble breathing, faint, or shaky in these situations. | 174 | 31.2 | 233 | 41.8 | 73 | 13 | 45 | 8 | 33 | 6 |
| 5 | Felt tense muscles, felt on edge or restless, or had trouble relaxing due to Corona. | 146 | 26.2 | 215 | 38.5 | 104 | 18.6 | 53 | 9.5 | 40 | 7.2 |
| 6 | Avoided, or did not approach or enter, situations linked with Corona. | 54 | 9.7 | 76 | 13.6 | 164 | 29.4 | 153 | 27.4 | 111 | 19.9 |
| 7 | Moved away from these situations or left them early. | 47 | 8.4 | 84 | 51 | 155 | 27.8 | 179 | 32 | 93 | 16.7 |
| 8 | Spent a lot of time preparing for, or procrastinating about (i.e., putting off), situations linked to Corona. | 69 | 12.4 | 143 | 25.6 | 161 | 28.9 | 113 | 20.3 | 72 | 12.9 |
| 9 | Distracted myself to avoid thinking about Corona. | 117 | 21 | 181 | 32.4 | 105 | 18.8 | 93 | 16.7 | 62 | 11 |
| 1 | Needed help to cope with these situations (e.g., alcohol or medications, superstitious objects, other people). | 122 | 21.9 | 181 | 32.4 | 128 | 22.9 | 94 | 16.8 | 33 | 5.9 |

**Appendix C. Response rate to the items in the Fear of COVID-19 Scale (7-items).**

| **N** | **Item**  . | **Strongly agree** | | **Agree** | | **Not sure** | | **Disagree** | | **Strongly disagree** | |
| --- | --- | --- | --- | --- | --- | --- | --- | --- | --- | --- | --- |
|  |  | **N** | **%** | **N** | **%** | **N** | **%** | **N** | **%** | **N** | **%** |
| 1 | I am most afraid of coronavirus. | 135 | 24.2 | 200 | 35.8 | 129 | 23 | 62 | 11 | 32 | 7.5 |
| 2 | It makes me uncomfortable to think about coronavirus. | 112 | 20 | 165 | 29.6 | 151 | 27 | 92 | 16.5 | 38 | 6.8 |
| 3 | My hands become clammy when I think about coronavirus. | 216 | 38.7 | 262 | 47 | 45 | 8 | 22 | 3.9 | 13 | 2.3 |
| 4 | I am afraid of losing my life because of coronavirus | 174 | 31.2 | 207 | 37 | 102 | 18.3 | 49 | 8.8 | 26 | 4.7 |
| 5 | When watching news and stories about coronavirus-19 on social media, I become nervous or anxious. | 109 | 19.5 | 137 | 24.6 | 177 | 31.7 | 90 | 16 | 45 | 8 |
| 6 | I cannot sleep because I am worrying about getting coronavirus. | 245 | 43.9 | 229 | 41 | 48 | 8.6 | 21 | 3.8 | 15 | 2.7 |
| 7 | My heart races or palpitates when I think about getting coronavirus. | 204 | 36.6 | 215 | 38.5 | 86 | 15.4 | 33 | 5.9 | 20 | 3.6 |
